# COVID-19: What Iodine Maps From Perfusion CT can reveal—A Prospective Cohort Study

**DOI:** 10.1186/s13054-020-03333-3

**Published:** 2020-10-21

**Authors:** Mario G. Santamarina, Dominique Boisier Riscal, Ignacio Beddings, Roberto Contreras, Martiniano Baque, Mariano Volpacchio, Felipe Martinez Lomakin

**Affiliations:** 1 Radiology Department, Hospital Naval Almirante Nef, Subida Alesandri S/N., Viña del Mar, Provincia de Valparaíso Chile; 2 Radiology Department, Hospital Dr. Eduardo Pereira, Valparaiso, Chile; 3 Intensive Care Unit, Hospital Naval Almirante Nef, Viña del Mar, Chile; 4 Radiology Department, Clinica Bupa Santiago, Santiago, Chile; 5 Intensive Care Unit, Hospital San Martin de Quillota, Quillota, Chile; 6 Intensive Care Unit, Hospital IESS Los Ceibos, Guayaquil, Ecuador; 7 Radiology Department, Centro de Diagnóstico Dr. Enrique Rossi, Buenos Aires, Argentina; 8 Viña del Mar, Escuela de Medicina, Facultad de Medicina Viña del Mar, Universidad Andres Bello, Valparaiso, Chile

**Keywords:** COVID-19, Coronavirus, Computed tomography angiography, Angiotensin converting enzyme 2, Angiotensin II, Vasoconstriction, Vasoplegia, Ventilation-perfusion ratio

## Abstract

**Background:**

Subtraction CT angiography (sCTA) is a technique used to evaluate pulmonary perfusion based on iodine distribution maps. The aim of this study is to assess lung perfusion changes with sCTA seen in patients with COVID-19 pneumonia and correlate them with clinical outcomes.

**Material and methods:**

A prospective cohort study was carried out with 45 RT-PCR-confirmed COVID-19 patients that required hospitalization at three different hospitals, between April and May 2020. In all cases, a basic clinical and demographic profile was obtained. Lung perfusion was assessed using sCTA. Evaluated imaging features included: Pattern predominance of injured lung parenchyma in both lungs (ground-glass opacities, consolidation and mixed pattern) and anatomical extension; predominant type of perfusion abnormality (increased perfusion or hypoperfusion), perfusion abnormality distribution (focal or diffuse), extension of perfusion abnormalities (mild, moderate and severe involvement); presence of vascular dilatation and vascular tortuosity. All participants were followed-up until hospital discharge searching for the development of any of the study endpoints. These endpoints included intensive-care unit (ICU) admission, initiation of invasive mechanical ventilation (IMV) and death.

**Results:**

Forty-one patients (55.2 ± 16.5 years, 22 men) with RT-PCR-confirmed SARS-CoV-2 infection and an interpretable iodine map were included. Patients with perfusion anomalies on sCTA in morphologically normal lung parenchyma showed lower Pa/Fi values (294 ± 111.3 vs. 397 ± 37.7, *p* = 0.035), and higher D-dimer levels (1156 ± 1018 vs. 378 ± 60.2, *p* < 0.01). The main common patterns seen in lung CT scans were ground-glass opacities, mixed pattern with predominant ground-glass opacities and mixed pattern with predominant consolidation in 56.1%, 24.4% and 19.5% respectively. Perfusion abnormalities were common (36 patients, 87.8%), mainly hypoperfusion in areas of apparently healthy lung. Patients with severe hypoperfusion in areas of apparently healthy lung parenchyma had an increased probability of being admitted to ICU and to initiate IMV (HR of 11.9 (95% CI 1.55–91.9) and HR 7.8 (95% CI 1.05–61.1), respectively).

**Conclusion:**

Perfusion abnormalities evidenced in iodine maps obtained by sCTA are associated with increased admission to ICU and initiation of IMV in COVID-19 patients.

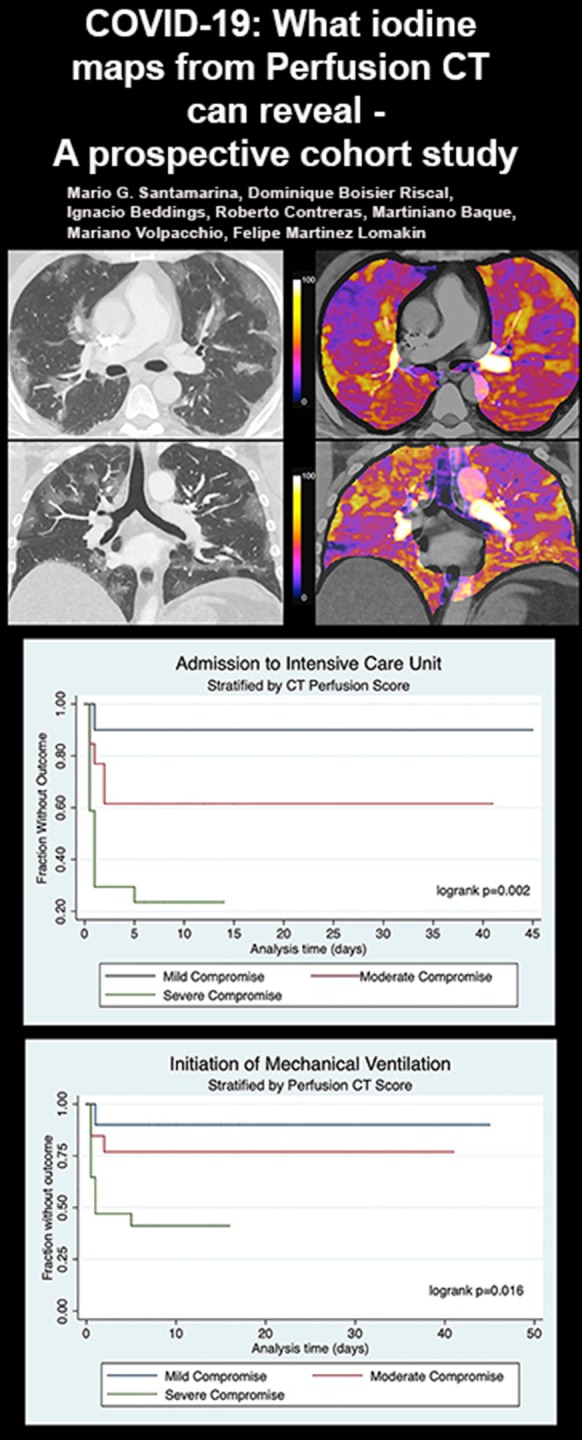

## Background

In December 2019, a novel human coronavirus, SARS-CoV-2, was reported in the city of Wuhan, China. It has spread throughout the world and caused a pandemic coronavirus disease (COVID-19). As of 22th July 2020, the disease had affected more than 15 million people causing more than 620,000 deaths, with the United States becoming the latest epicenter with near 3,960,000 cases and the highest number of deaths—over 140,000 people [1].

SARS-CoV-2 infection begins with inoculation of the respiratory tract mucosa using angiotensin-converting enzyme 2 (ACE2) as the functional receptor for cell entry. The inhaled virus likely binds to epithelial cells in the nasal cavity and starts replicating. There is local propagation of the virus, with a limited initial innate immune response. At this stage the virus can be detected via nasal swabs, and although viral burden may be low, these individuals are contagious. The virus propagates and migrates down the respiratory tract along the conducting airways, and a more robust innate immune response is triggered. For about 80% of infected patients, the disease will be mild and mostly restricted to the upper and conducting airways. However, the remaining 20% of infected patients will progress and have severe pulmonary infiltrates, while some of these will develop very severe disease and eventually die [2].

A wealth of scientific data has been published over the past few months on epidemiology, clinical manifestations, molecular biology, imaging features, laboratory diagnosis and treatments, but evidence has not been robust in most literature. Although COVID-19 pneumonia may sometimes meet the ARDS Berlin definition criteria, some authors argue that it is a specific disease with peculiar phenotypes, due to the frequent dissociation between the severity of hypoxemia and the maintenance of relatively good respiratory mechanics [3].

Imaging plays a crucial role in the initial detection and evaluation of progression of COVID-19 pneumonia.

To the best of our knowledge, there is currently little scientific data regarding lung perfusion disturbances occurring in patients with COVID-19 pneumonia [4]. Our goal is to assess lung perfusion changes seen in patients with COVID-19 pneumonia, and to correlate perfusion abnormalities with clinical outcomes.

## Materials and methods

### Patient cohort

This study was approved by our Institutional Review Board at the Hospital Naval Almirante Nef and written informed consent was waived. A prospective cohort study was undertaken to assess the prognostic ability of lung perfusion using subtraction CT angiography (sCTA). Patients (> 18 years old) with confirmed SARS-CoV-2 infection by real-time reverse transcription polymerase chain reaction (RT-PCR) that required hospitalization were consecutively enrolled at three hospitals between April and May 2020. These centers included the Hospital Naval Almirante Nef (Viña del Mar, Chile), Hospital San Martín de Quillota (Quillota, Chile), and Hospital IESS Los Ceibos (Guayaquil, Ecuador). These facilities are tertiary-care hospitals able to provide on-site CTA scans and intensive care management of severe COVID-19 cases when needed.

Forty-five patients were included in this study. However, due to uninterpretable iodine map results, four of these participants had to be excluded from analyses. This left 41 patients (22 males and 19 females) in the study. In all cases, a basic clinical and demographic profile was obtained that included information regarding sex, age, duration of clinical symptoms, and laboratory studies including D-dimer, alveolar oxygenation to inspired oxygen (Pa/Fi) ratio at baseline and sequential organ failure assessment (SOFA) score. Baseline lung CT characteristics were recorded as well such as the predominant imaging pattern (ground-glass opacities, consolidation or mixed pattern), the extension of involved lung, presence of pleural effusion, evidence of right-ventricular overload and pulmonary embolism. These data were recorded along with sCTA features (see below) in an anonymized registry.

All participants were followed-up until hospital discharge searching for the development of any of the study endpoints. These endpoints included intensive-care unit (ICU) admission, initiation of invasive mechanical ventilation (IMV) and death. The decision to intubate or admit to an ICU facility was left to the attending physician's discretion.

### CT scan protocol and image acquisition

All CT scans were performed within 24 h of admission to the hospital, in supine position. Imaging data were acquired with multidetector-CT (Canon Aquilion Prime 160 and 80, and Canon Aquilion RXL 16).

### CT scan protocol

After positioning, an unenhanced scan was obtained, followed by IV injection of 100 mL iodinated contrast medium at a rate of 5 mL/seg (Visipaque 320, GE Healthcare, Milwaukee, Wis and Optiray 320, Mallinckrodt Medical, St Louis, Mo). After bolus triggering at the level of the pulmonary artery with a relative threshold of 150 HU, an early pulmonary arterial angiographic phase was obtained, followed eight seconds later by a delayed pulmonary arterial phase. All the exams were performed with the same acquisition parameters: kV (100 kV), automatic exposure control (Standard), similar range and field-of-view (FOV: L or LL), collimation (1.0 × 16 for Canon Aquilion RXL 16; 0.5 × 80 for Canon Aquilion Prime 80 and 160), pitch factor (1.4), and same rotation speed (0.5 s for Canon Aquilion RXL 16 and 0.35 s for Canon Aquilion Prime 80 and 160). The acquired images had the same reconstruction parameters: slice thickness (1.0 mm), reconstruction interval (0.8 mm) and convolution filter (mediastinum). Average dose DLP (mGy/cm) was 1093 (range: 847–1932). Breathing instructions were the same for all examinations.

sCTA is a technique that uses software-based motion correction between an unenhanced and an enhanced CT scan to obtain an iodine distribution map of the lung parenchyma. Iodine distribution maps of the early and delayed arterial phases were obtained using SureSubtraction software (version 7.0; Canon Medical Systems, Japan). Iodine maps were generated with gray-scale and identical color window tables, ranging from blue (low iodine enhancement) to yellow (high iodine enhancement).

### Image analysis

Areas of injured parenchyma in both lungs were assessed for a predominant pattern. These were characterized as ground glass opacities, consolidation and mixed pattern. Airspace compromise was assessed for each of the 5 lobes considering the extent of anatomic involvement, as follows: 0 points for no involvement; 1 point for < 30% involvement; 2 points for 31–60% involvement; and 3 points for > 61% involvement. The resulting CT severity score was the sum of each individual lobar score (0–15). Areas of airspace disease were also assessed for the presence of increased perfusion or hypoperfusion. Peripheral vascular dilatation and vascular tortuosity were also assessed in each lobe.

Areas of apparently healthy lung parenchyma were assessed for preservation of anteroposterior and apicobasal perfusion gradient or hypoperfusion. Hypoperfusion in apparently normal lung parenchyma was characterized according to its distribution (focal or diffuse), and extension. The latter was graded using a scoring system: first, both lungs were divided into five lobes (upper right and left lobes, middle lobe, and lower right and left lobes). Then, the extension of hypoperfusion in apparently healthy lung parenchyma in each lobe was categorized as normal perfusion (0 points), less than 50% of the lobe affected (1 point), and 50% of the lobe or more affected (2 points). This resulted in an overall score that ranged from 0 to 10 points, with higher scores indicating more severe hypoperfusion. Using this score, patients were divided in three groups. Patients with 3 points or less were considered to have mild perfusion abnormalities, those with 4–6 points had moderate abnormalities and those with 7 or more points had severe abnormalities.

A single radiologist with over 15-years experience (MS) in reading CT analyzed the images of included patients in all hospitals. The attending physicians did not have information regarding sCTA perfusion results. However, data regarding overall CT characteristics of included participants was made available to clinicians as per current protocols at study centers.

### Statistical analyses

Descriptive statistics including medians, means, standard deviations, interquartile ranges (IQR) and absolute and relative frequencies were used first to describe the study sample. Bivariate comparisons between groups were performed using Fisher's Exact test for categorical variables and Student's t test or Mann–Whitney's test for continuous variables after reviewing data distribution and variances. Association between quantitative variables were sought using Pearson's or Kendall's correlation coefficients. Discrete quantitative variables (such as computed tomography scores) were evaluated using Kendall's tau coefficient. The development of any of the aforementioned study endpoints was evaluated using Kaplan–Meier survival curves which were then compared using the *logrank* statistic. Ninety-five percent confidence intervals were calculated whenever appropriate. Analyses were undertaken by an independent statistician who had no participation in the decision to admit or intubate any of the included patients, using STATA 15.0 SE® (College Station, TX: StataCorp LLC). A two-sided *p* value of < 5% was considered to be statistically significant.

## Results

### Patient characteristics

All patients had SARS-CoV-2 infection confirmed by a positive RT-PCR. The mean age was 55.2 ± 16.5 years and the median duration of symptoms was 7 (IQR 5–8) days. Laboratory investigations were often abnormal. Twenty-three patients (59%) had abnormal D-dimer levels, which averaged 1057 ± 984.1 ng/mL. The average Pa/Fi ratio on admission was 307 ± 110.2 and the median SOFA score was 2 (IQR 1–2) points, which was similar between study groups (*p* = 0.09). Patients with hypoperfusion abnormalities on apparently healthy lung parenchyma on sCTA showed lower Pa/Fi values (294 ± 111.3 vs. 397 ± 37.7, *p* = 0.035), and higher D-dimer levels (1156 ± 1018 vs. 378 ± 60.2, *p* < 0.01). A summary of baseline clinical characteristics is shown in Table 1.

**Table 1:** Baseline characteristics

Characteristic	Normal perfusion CT (n = 5)	Abnormal perfusion CT (n = 36)	Total (n = 41)	*p* value
*Clinical characteristics*				
Mean age (years) (SD)	46.8 ± 11.5	56.4 ± 16.8	55.2 ± 16.5	0.28^a^
Male sex (n, %)	1 (20%)	21 (58.3%)	22 (53.7%)	0.16^b^
Median SOFA score (IQR)	1 (1)	2 (1–3)	2 (1–2)	0.09^b^
Median duration of symptoms (IQR)	6 (4–7)	7 (5–8)	7 (5–8)	0.44^a^
Mean Pa/Fi ratio (SD)	397 ± 37.7	294 ± 111.3	307 ± 110.2	0.035^a^
Mean D-Dimer levels (ng/mL) (SD)	378 ± 60.2	1156 ± 1018.1	1057 ± 984.1	< 0.01
*Computed tomography findings*				
Predominant pattern (n, %)				
Ground-glass opacities	5 (100%)	18 (50%)	23 (56.1%)	0.21^b^
Mixed pattern with predominant ground-glass opacities	0 (0%)	10 (27.8%)	10 (24.4%)	
Mixed pattern with predominant consolidation	0 (0%)	8 (22.2%)	8 (19.5%)	
Median number of compromised lobes (IQR)	4 (2–5)	5(5)	5(5)	0.02^a^
Pleural effusion (n, %)	2 (40%)	5 (13.9%)	7 (17.1%)	0.20^b^
Pulmonary embolism (n, %)	0 (0%)	2 (5.6%)	2 (4.8%)	1.0^b^
Right ventricular overload (n, %)	0 (0%)	0 (0%)	0 (0%)	–
Vascular dilatation (n, %)	4 (80%)	36 (100%)	40 (97.5%)	0.12^b^
Median number of lobes with vascular dilatation (IQR)	2 (2)	3.5 (2–5)	3 (2–5)	0.17^a^
Vascular tortuosity (n, %)	0 (0%)	14 (38.9%)	14 (34.2%)	0.15^b^
Median CT severity score (IQR)	3 (2–5)	7 (5–10)	7 (5–10)	0.01^b^

*SD* standard deviation, *IQR* interquartile range, *SOFA* sequential organ failure assessment, *CT* computed tomography

^a^Mann–Whitney *U* test

^b^Fisher’s exact test

### Lung CT evaluation

The most common patterns seen in lung CT scans were ground-glass opacities (23 patients, 56.1%), followed by mixed pattern with predominant ground-glass opacities (10 patients, 24.4%) and mixed pattern with predominant consolidation (8 patients, 19.5%).

There were no major differences in the predominant radiological pattern between normal and abnormal perfusion groups. Multilobar lung involvement was common among included patients but tended to be less among patients without perfusion abnormalities (*p* = 0.02). In the same way, the median CT severity score was higher and statistically significant in the group with perfusion abnormalities (7 (IQR 5–10) vs. 3 (IQR 2–5), *p* = 0.01).

**Fig. 1.**
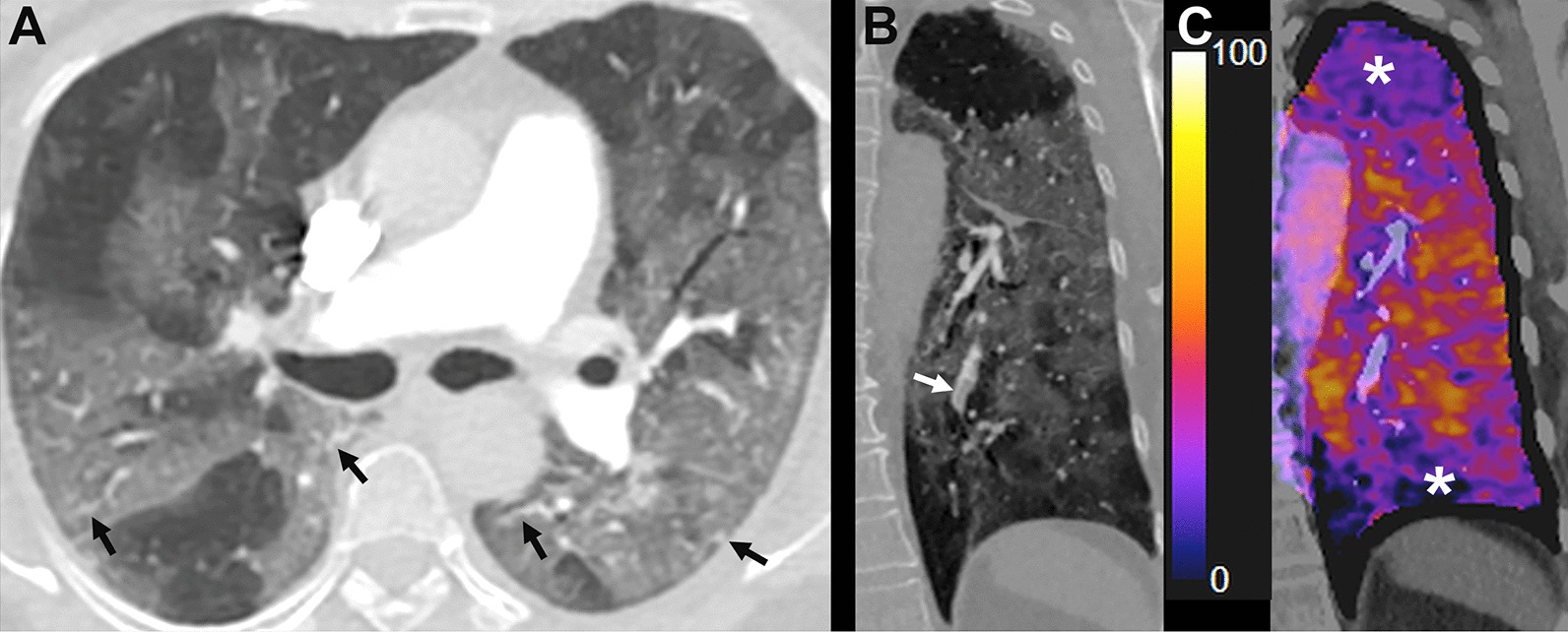
78-year-old female patient, RT-PCR-confirmed COVID-19, 8 days since symptom onset, complicated with pulmonary embolism. Admission PaO_2_/FiO_2_ ratio was 162, and d-dimer levels > 2000 ng/mL. Admitted to the intensive care unit, managed with invasive mechanical ventilation. She died 2 weeks after admission. **a** Axial lung-window CT angiography image shows extensive lung involvement with patchy ground-glass opacities in both lungs, with vascular dilatation in small peripheral subsegmental pulmonary arterial branches, some of them with a varicose appearance (black arrows). **b** Coronal CT angiography image shows pulmonary embolism in the posterior basal segment of the lower left lobe (white arrow). **c** 5 mm coronal-plane reconstruction of a subtraction iodine map shows moderate to severe hypoperfusion in superior and inferior regions of the lung (*), in areas of apparently healthy lung parenchyma in conventional chest CT images, which is more pronounced in the posterior basal segment of the lower left lobe, in relation to the area of pulmonary embolism. Areas of ground-glass opacities show normal or increased perfusion, most probably due to vasoplegia

Pulmonary embolism was found in two patients (4.8%), and both had an abnormal perfusion CT. Pleural effusion was infrequent among included participants (7 patients, 17.1%). No patient showed signs of right ventricular overload at baseline. Vascular dilatation, defined as a 1.5-fold increase in arterial diameter compared to the accompanying bronchus, was observed in 40 (97.5%) patients. Vascular tortuosity was found in 14 patients, all belonging to the group with perfusion abnormalities. The median number of lobes with vascular dilatation was also higher in the group that showed perfusion anomalies. The presence of vascular tortuosity was significantly associated with the requirement of invasive mechanical ventilation (HR 5.9, 95% CI 1.83–18.91, *p* = 0.003) (Fig. 1).

### Perfusion findings and study outcomes

**Fig. 2.**
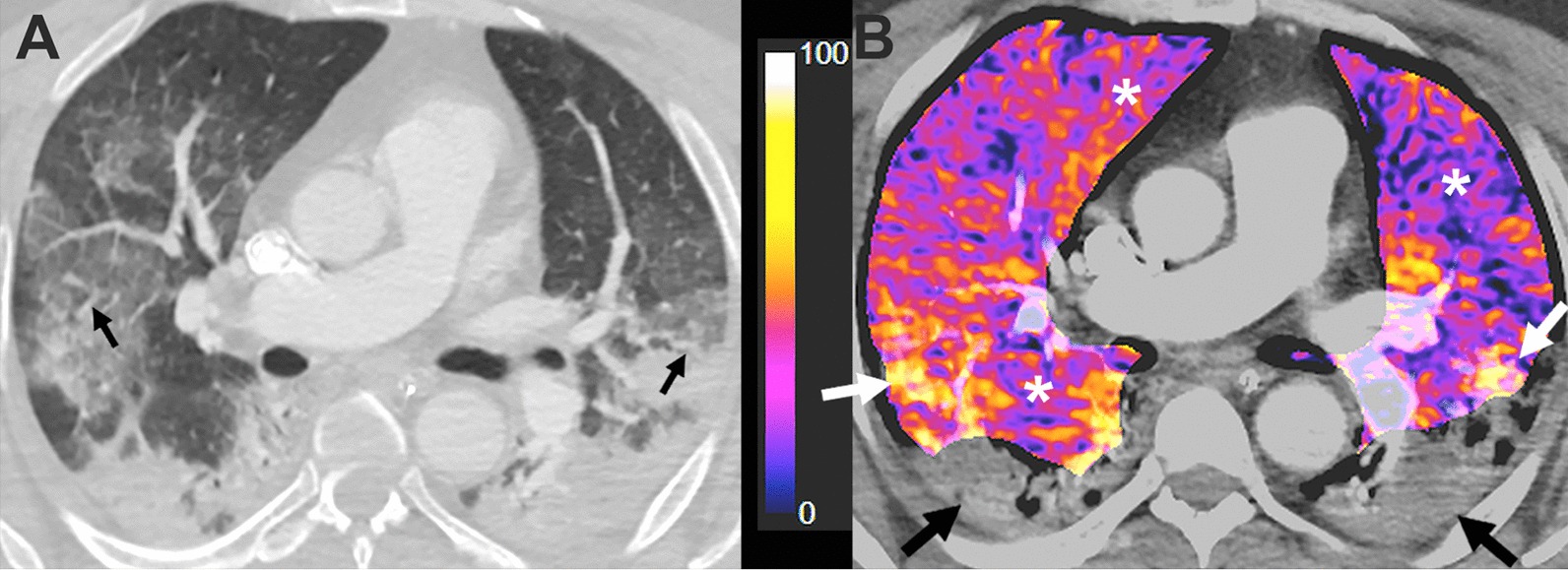
51-year-old male patient, RT-PCR confirmed COVID-19, 3 days since symptom onset. Admission PaO_2_/FiO_2_ ratio was 240, and d-dimer level was 480 ng/mL. Admitted to the intensive care unit, managed with invasive mechanical ventilation. **a** Axial lung-window CT angiography image shows extensive bilateral ground-glass opacities, with areas of posterior subpleural consolidation. There is subtle subsegmental peripheral vascular dilatation of pulmonary arterial branches (small black arrows). **b** 5 mm axial reconstruction of a subtraction iodine map shows slight to moderate hypoperfusion predominantly in areas of non-injured lung (*), and more prominent areas of increased perfusion in relation to the zones of ground-glass opacities (white arrows). Perfusion was not assessed in the areas of consolidation (black arrows) due to software limitations that exclude these zones. This patient evolved in a similar fashion to the H (type 2) phenotype described by Gattinoni et al.

**Fig. 3.**
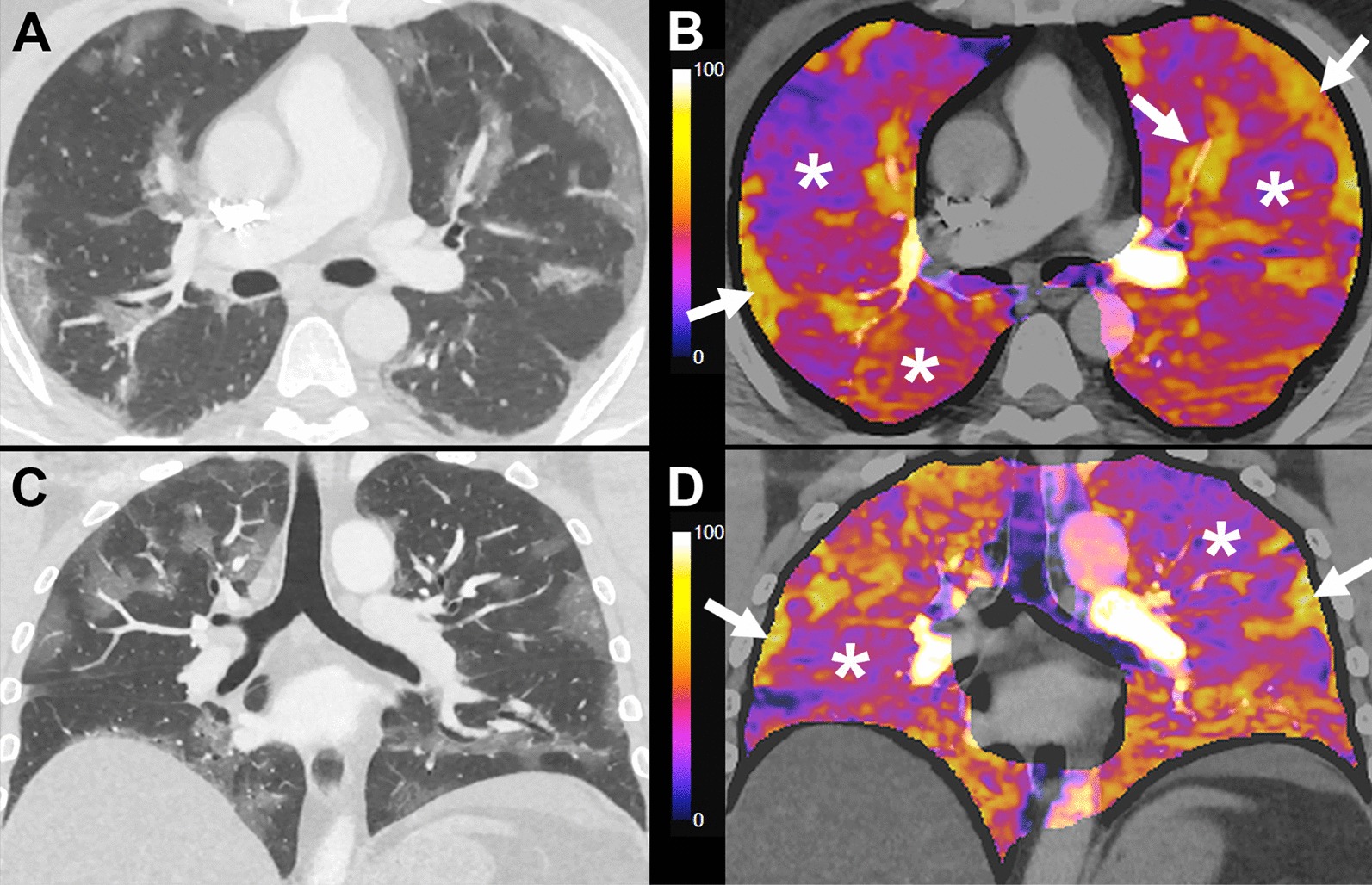
37-year-old male patient, RT-PCR-confirmed COVID-19, 10 days since symptom onset. Admission PaO_2_/FiO_2_ ratio was 240 and d-dimer level was 480 ng/mL. Admitted to the intensive care unit, managed with invasive mechanical ventilation. **a**, **c** Axial and coronal lung-window chest CT angiography images show multiple foci of ground-glass opacities, with a predominantly subpleural distribution, with areas of apparently healthy lung parenchyma. Vascular dilatation can be seen in relation to areas of ground-glass opacities. **b**, **d** 5 mm reconstruction images of subtraction iodine maps in corresponding axial and coronal planes, show areas of severe hypoperfusion in the corresponding apparently healthy lung parenchyma (*), with increased perfusion in areas of ground-glass opacities (white arrows). This patient evolved in a similar fashion to the L (type 1) phenotype described by Gattinoni et al.

Perfusion abnormalities were common among included participants with 36 (87.8%) patients having hypoperfusion in areas of non-injured parenchyma. These anomalies had most commonly a diffuse distribution (23 patients, 63.9%). The median perfusion-CT score for areas of decreased perfusion was 5 points (IQR 3–9). Patients were then categorized in three groups using the aforementioned scoring system according to the extension of perfusion abnormalities in non-injured lung. Eleven patients (26.8%) were identified as having mild perfusion anomalies, 13 patients (31.7%) had moderate perfusion abnormalities and 17 (41.5%) had severe abnormalities (Figs. 2, 3).

**Fig. 4:**
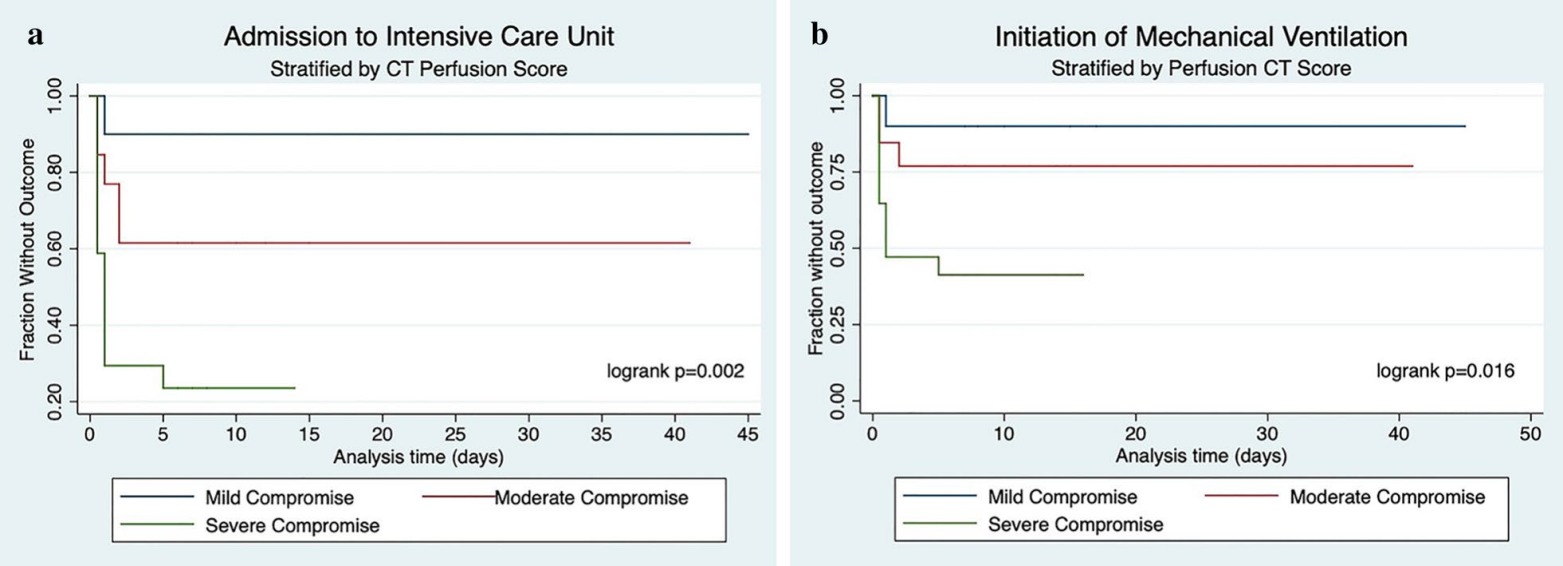
**a** Admission to Intensive Care Unit. Stratified by CT Perfusion Score. **b** Initiation of Mechanical Ventilation. Stratified by CT Perfusion Score

**Fig. 5.**
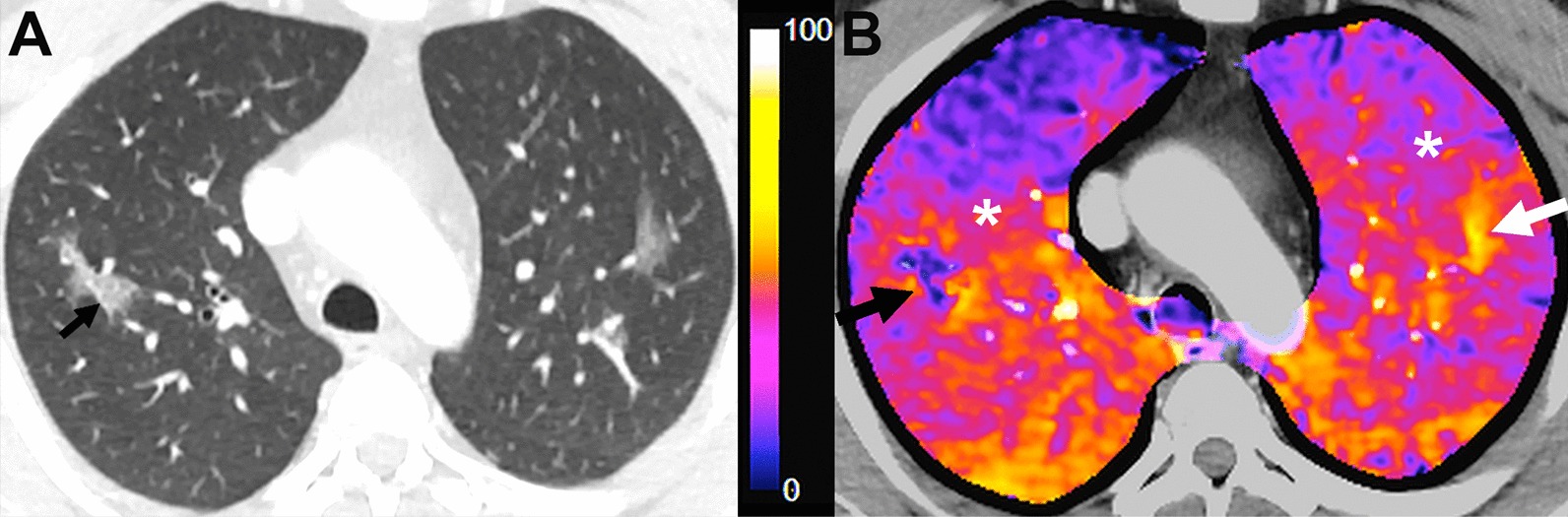
40-year-old male patient, RT-PCR-confirmed COVID-19, 5 days since symptom onset. Admission PaO_2_/FiO_2_ ratio was 310, and d-dimer levels < 300 ng/mL. Due to progressive hypoxaemia, he was managed in the intensive care unit with conscious prone position and high flow nasal cannula, with positive tolerance and evolution. **a** Axial lung-window CT angiography image shows small patchy areas of ground-glass opacities, with slightly dilated small pulmonary arterial branches (small black arrow). **b** 5 mm axial reconstruction of a subtraction iodine map shows moderate hypoperfusion with a right-sided predominance (*). The ground-glass opacity in the upper right lobe shows decreased perfusion within the opacity, with a peripheral halo of increased perfusion (black arrow). These findings could be explained by physiological hypoxic vasoconstriction. However, the ground-glass opacity in the upper left lobe shows increased perfusion (white arrow)

**Fig. 6:**
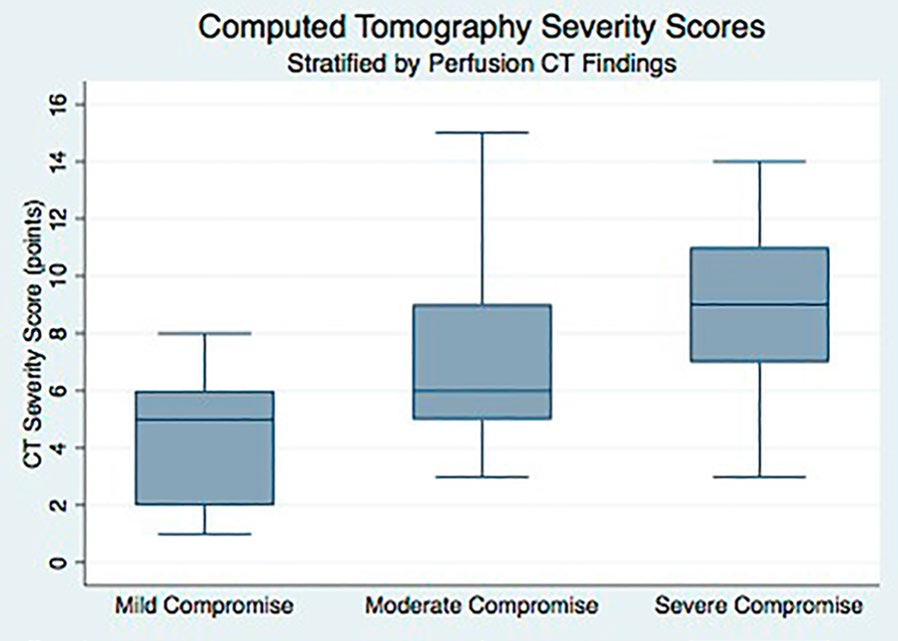
Computed Tomography Severity Scores stratified by Perfusion CT Findings

Nineteen patients (46.3%) were admitted to the ICU during follow up, and 14 (34.2%) required IMV. Two patients (4.9%) died during hospitalization. None of the patients with normal perfusion CT scans required admission to the ICU or IMV, and all survived. Survival analyses showed significant differences in the probability of ICU admission and initiation of IMV between perfusion score groups, which are shown in Fig. 4a, b. Patients with severe hypoperfusion in apparently healthy lung parenchyma had a greatly increased probability of being admitted to the ICU, with a HR of 11.9 (95% CI 1.55–91.9, *p* = 0.017) when contrasted with patients with mild perfusion abnormalities. Similar findings were seen regarding the initiation of IMV (HR 7.8 95% CI 1.05–61.1, *p* = 0.025). Patients with moderate hypoperfusion anomalies also had an increased risk of ICU admission and of requiring IMV, albeit without statistical significance (HR 4.5 95% CI 0.53–38.4, *p* = 0.17 and HR 2.60, 95% CI 0.27–25, *p* = 0.41, respectively). A summary of study outcomes is shown in Table 2. Thirty-four of these patients (94.4%) showed increased perfusion to areas with visible parenchymal abnormalities, with the remaining two patients showing perfusion defects in these zones (5.6%) as their predominant finding (Fig. 5). 14 (34%) patients had mild artifacts on iodine maps that did not interfere with the analysis of the images. A moderate correlation was found between perfusion CT score and CT severity score (TauB = 0.47), which was statistically significant (*p* < 0.001). This association is shown in Fig. 6.

**Table 2:** Study outcomes

Outcome	Mild compromise (n = 11)	Moderate compromise (n = 13)	Severe compromise (n = 17)	*p* value^a^
Admission to intensive care unit (n, %)	1 (9.1%)	5 (38.5%)	13 (76.5%)	0.002
Initiation of mechanical ventilation (n, %)	1 (11.1%)	3 (23.1%)	10 (58.8%)	0.016
Death (n, %)	0 (0%)	1 (7.7%)	1 (5.9%)	0.72

^a^Logrank statistic

## Discussion

With the advent of SARS-Cov-2, nearly 20% of the infected patients will progress and develop severe disease, consistent in acute hypoxemic respiratory failure, and eventually die [2]. In our prospective cohort, we evaluated and quantified lung perfusion disturbances in patients with COVID-19 pneumonia as a severity predictor, through iodine distribution maps obtained with sCTA. Our findings showed that with increasing severity of hypoperfusion abnormalities in areas of apparently healthy lung parenchyma in conventional chest CT images, patients had a statistically significant increase in their chance to require admission to ICU (*p* = 0.002), and to require IMV (*p* = 0.016). Vascular tortuosity in CT angiography images was also independently associated with requirement of IMV (*p* = 0.003). We also found that average lower Pa/Fi ratio (*p* = 0.035), higher D-dimer levels (*p* < 0.01), and number of affected lung lobules (*p* = 0.02), were associated with perfusion abnormalities.

Patients with COVID-19 pneumonia showed abnormally decreased iodine distribution in areas of apparently normal lung parenchyma, findings that as a whole probably account for a severe V/Q mismatch. Some authors have suggested microthrombi to be a key component of clinical deterioration. Activation of the coagulation cascade in early stages of disease appears to be due to a thromboinflammatory response and direct viral effects on pulmonary tissue, which is counter-balanced by fibrinolytic activity and manifests as an increase in fibrin degradation products. In later stages of disease, this balance is lost and a pro-coagulant state ensues [5–8].

ACE2 dysfunction could be a possible explanation for the establishment of a systemic endotheliopathy that may lead to an abnormal pro-coagulant state and sepsis [9]. We believe that the main pathophysiologic mechanism behind COVID-19 pneumonia involves accumulation of angiotensin II due to decreased activity of ACE2, which is secondary to ACE2 endocytosis after viral binding [10]. This leads to vasoconstriction, establishing a progressive V/Q mismatch, with extensive areas of apparently healthy but hypoperfused lung that function as alveolar dead space.

This is supported by studies showing evidence that non-hypertense patients with COVID-19 appeared to have elevated levels of plasma angiotensin II, which were correlated with the degree of lung injury and total viral load [11, 12].

Decreased activity of ACE2 leads to heightened and relatively unopposed vasoconstriction, pro-coagulation, pro-inflammatory and pro-oxidant angiotensin II effects [13, 14].

In a scenario of extensive vasoconstriction in well-ventilated areas, possible treatment strategies could be aimed at improving perfusion. Luni Chen et al. treated patients with severe SARS in 2003 with inhaled nitric oxide, with significant improvement in arterial oxygenation [15].

Areas of injured lung parenchyma characterized by ground-glass opacities, consolidation and septal thickening, also have particular anomalies in COVID-19 pneumonia. Subsegmental vessel enlargement in the vicinity of areas of injured parenchyma has been described in up to 89% of patients with COVID-19 pneumonia [2]. Although in situ microthrombosis is a possible underlying mechanism, it could also be explained by vasoplegia induced by SARS-CoV-2. Overactivation of a regional vasodilatation cascade in areas of injured non-aerated lung parenchyma, due to a local dysfunctional inflammatory response, could explain the loss of the normal, physiological hypoxic arterial vasoconstriction that would normally be expected in areas of hypoventilated lung, contributing to the abnormal hyperperfusion that we have found in areas of lung opacities.

Gattinoni et al. [3] hypothesized that there are two distinct types of lung damage in ARDS. Type-L or 1 is characterized by low elastance and high compliance, which is rarely seen in ARDS, while type-H or 2 shows high elastance and low compliance that is typical of ARDS. They explain the primary cause of hypoxemia in type-L is perfusion defects presumably caused by vasoconstriction and high shunt fraction. In contrast, the high elastance in type-H is thought to be induced by lung edema.

In type-L pattern, areas of injured lung parenchyma show hyperperfusion, probably due to loss of compensatory hypoxic pulmonary vasoconstriction (vasoplegia), resulting in high perfusion to areas of hypoventilated lung and an abnormally low V/Q ratio; while areas of apparently normal lung parenchyma show hypoperfusion that results in an abnormally high V/Q ratio. Changes in vascular resistance lead to a net shunt or steal of vascular flow towards areas of non-aerated lung, which is supported by the good clinical response to prone position that has been described in patients with COVID-19 [16].

This study has several limitations. First, the small sample size, mainly explained by the epidemiologic situation of the cities where the participant hospitals are located at the moment of the study, which were in early stages of the pandemic outbreak. Our relatively small sample size restricted the use of multivariable techniques to further refine the association with perfusion CT findings and prognosis. Although the bivariate contrasts were both clinically and statistically significant, it is not possible to exclude bias due to confounding, especially considering the correlation shown in Fig. 6. However, it should also be kept in mind that the overall strength of the correlation was only moderate. This reduces the possibility that our findings are explained solely by confounding. Further studies with larger sample sizes are needed to fully assess this association.

Second, the analysis of the images was performed by a single radiologist, resulting in a lack of inter-observer variability assessment. Third, perfusion imaging involves more radiation than conventional CT techniques. Fourth, dual-energy CT (DECT) is commonly used in clinical practice for assessing pulmonary perfusion and uses material decomposition of iodine from other materials to visualize the regional distribution of intravenous contrast in the pulmonary vessels, including the capillaries [17, 18]. Although sCTA is a new postprocessing technique and thus, has not been as extensively validated as DECT, it is a promising approach that digitally subtracts a precontrast CT scan from a contrast material–enhanced CT scan after motion correction. Since it is software-based, it is significantly less expensive and potentially more available than costly DECT equipment. Nevertheless, being a novel approach, concerns regarding potential bias resulting from subtraction of non-contrast images from contrast-enhanced images need to be addressed, and greater experience and better knowledge of typical pitfalls is needed to improve the diagnostic accuracy [19–21]. Fifth, some of the iodine maps were uninterpretable. Finally, it is unclear whether these perfusion abnormalities are unique to COVID-19 or if they can also be found in other multifocal pneumonias and other causes of ARDS.

## Conclusions

Severe lung perfusion abnormalities, evaluated by iodine maps, were associated with a higher ICU admission and initiation of IMV. Perfusion abnormalities were detected in both damaged and normal lung parenchyma, allowing us to state that different types of V/Q mismatch occur in the entire lung parenchyma in COVID-19 patients. These findings suggest considering the evaluation of therapies that are oriented towards improving pulmonary perfusion in COVID-19 and maybe could contribute to the management and outcome of these patients. The prognostic implications of hypoperfusion in normal lung parenchyma remain to be assessed independently from severity and extension of lung opacities.

## Data Availability

The datasets used and/or analyzed during the current study are available from the corresponding author on reasonable request.

## Abbreviations

COVID-19: Coronavirus Disease 2019
ACE2: Angiotensin-converting enzyme 2
sCTA: Subtraction CT angiography
ICU: Intensive Care Unit
IMV: Invasive mechanical ventilation
